# Predictive Biomarkers of Age-Related Macular Degeneration Response to Anti-VEGF Treatment

**DOI:** 10.3390/jpm11121329

**Published:** 2021-12-08

**Authors:** Ana I. Oca, Álvaro Pérez-Sala, Ana Pariente, Rodrigo Ochoa, Sara Velilla, Rafael Peláez, Ignacio M. Larráyoz

**Affiliations:** 1 Biomarkers and Molecular Signaling Group, Center for Biomedical Research of La Rioja (CIBIR), Foundation Rioja Salud, 26006 Logroño, La Rioja, Spain; aioca@riojasalud.es (A.I.O.); aperez@riojaslud.es (Á.P.-S.); apariente@riojaslud.es (A.P.); rochoaf@riojasalud.es (R.O.); svelillaoses@gmail.com (S.V.); rpelaez@riojasalud.es (R.P.); 2 Unidad Predepartamental de Enfermería, Universidad de La Rioja (UR), 26006 Logroño, La Rioja, Spain

**Keywords:** RNA-Seq, PBMC, retina, ranibizumab, machine learning

## Abstract

Age-related macular degeneration (AMD) is an incurable disease associated with aging that destroys sharp and central vision. Increasing evidence implicates both systemic and local inflammation in the pathogenesis of AMD. Intravitreal injection of anti-vascular endothelial growth factor (VEGF) agents is currently the first-line therapy for choroidal neovascularization in AMD patients. However, a high number of patients do not show satisfactory responses to anti-VEGF treatment after three injections. Predictive treatment response models are one of the most powerful tools for personalized medicine. Therefore, the application of these models is very helpful to predict the optimal treatment for an early application on each patient. We analyzed the transcriptome of peripheral blood mononuclear cells (PBMCs) from AMD patients before treatment to identify biomarkers of response to ranibizumab. A classification model comprised of four mRNAs and one miRNA isolated from PBMCs was able to predict the response to ranibizumab with high accuracy (Area Under the Curve of the Receiver Operating Characteristic curve = 0.968), before treatment. We consider that our classification model, based on mRNA and miRNA from PBMCs allows a robust prediction of patients with insufficient response to anti-VEGF treatment. In addition, it could be used in combination with other methods, such as specific baseline characteristics, to identify patients with poor response to anti-VEGF treatment to establish patient-specific treatment plans at the first visit.

## 1. Introduction

Age-related macular degeneration (AMD) is an incurable disease associated with aging that destroys sharp and central vision, and therefore, it is a highly disabling disease. AMD affects the macula, a light-sensitive region at the back center of the retina, responsible for seeing objects in fine detail. There are two main types of AMD: dry and wet AMD. Both forms can occur in one or both eyes, neither of them produces pain, so they can go undetected until they produce marked changes in vision. Dry AMD, the more common form of macular degeneration (90% of total cases), is proposed to be a vascular-metabolic-inflammatory disease characterized by the accumulation of drusen (small yellowish deposits) [1,2]. Dry AMD does not usually cause total loss of central vision due to its slow progress. Wet AMD, however, is the more serious form of the disease and it represents 90% of the cases that progress to legal blindness. It is characterized by choroidal neovascularization (CNV) as a result of abnormally choroidal blood vessels growth through Bruch’s membrane into the macula, pushing aside the retinal pigment epithelium (RPE) and distorting its structure [1]. Moreover, 10 to 15 percent of adults with dry AMD will go on to develop wet AMD as a consequence of CNV [1]. This process eventually leads to blood and fluid leakage that can scar the macula and retina, which leads to a rapid and permanent loss of central vision [1].

CNV is stimulated by the induction of angiogenic growth factors, including VEGF and several interleukins (IL-6, IL-8, IL-18…). Clinical trials and laser-induced CNV in animal models have shown that antiangiogenic therapy targeting VEGF, such as bevacizumab, aflibercept or ranibizumab, can decrease disease progression [3]. However, there are a number of problems with current therapy options. The high rate of retreatment, for one, places a remarkable burden on both patients and retinal specialists, while the high cost of therapy is creating economic challenges for national healthcare systems that are already under strain. In addition, the optimal treatment regimen for specific subsets of patients has yet to be defined in clinical trials. Finally, gains in visual acuity do have a limit, and an important subset of patients with wet AMD does not appear to respond, whether functionally or anatomically, to current anti-VEGF therapy [4].

Increasing evidence implicates both systemic and local inflammation in the pathogenesis of AMD. For instance, the presence of inflammatory mediators of the complement cascade in drusen, macrophages in the choroid of eyes with AMD [5], or anti-retinal autoantibodies and inflammatory markers detected in the sera of patients with AMD [6]. Involvement of systemic inflammation in the disease is reflected by the association of single nucleotide polymorphisms (SNPs) in complement components [7,8,9,10], chemokine receptors [11,12], VEGF isoforms [13,14] and Toll-like receptor-3 [15]. Furthermore, perturbed macrophage function is thought to lead to the development of features resembling AMD in mice strains deficient in chemokine receptors or their ligands [16]. Thus, plasmatic solutes and PBMCs seem to have an important role in AMD physiopathology [17,18,19,20] and have been widely used to study biomarkers related to AMD [21,22,23,24,25].

Although knowledge of the genetic history and DNA sequences of an individual patient may be helpful for the studies of ancestry or a diagnosis of pre-existing adversities to certain drugs, it has relatively little effectiveness for molecular medicine in AMD [26,27,28,29,30] when compared to analyzing transcriptomes. To our knowledge, no direct transcriptome-wide analysis of white blood cells in relation to AMD response to ranibizumab treatment has been reported.

Predictive treatment response models are one of the most powerful tools for personalized medicine as they can predict which treatment would fit better on each patient, probability of success, etc. Therefore, the application of these models is very helpful to predict the optimal treatment for an early application on each patient [23].

In the present work, we have analyzed the transcriptome of PBMCs from 59 wet AMD patients, before undergoing ranibizumab treatment. Using correlation-based feature selection algorithms, we have identified a set of mRNAs and miRNAs to build classification models for the prediction of ranibizumab response, with high accuracy. This set of RNAs could help clinicians to identify patients with poor response to anti-VEGF treatment, at the first visit, and to establish improved patient-specific treatment plans.

## 2. Materials and Methods

### 2.1. Population and Group Characteristics

Patients were recruited, evaluated and the progression was followed by Ophthalmologists from the Service of the Hospital San Pedro, La Rioja, Spain. Signed informed consent forms were obtained from 59 untreated wet AMD patients older than 60 years. They were evaluated based on ophthalmoscopy, fluorescein angiography and optical coherence tomography (OCT) in the Department of Ophthalmology of Hospital San Pedro. Patients with severe systemic diseases, such as malignancies, active ischemic heart disease, uncontrolled diabetes or pulmonary disease, or autoimmune diseases were excluded from the study. Classification of patient response was based on Amoaku and collaborators [31]. Briefly, patients were considered good responders (good or partial morphological and/functional response) when the examination showed resolution of intraretinal fluid (IRF), subretinal fluid (SRF), retinal thickening and/or improvement of at least five ETDRS letters. Poor responders (poor or no response) were defined as patients showing less than 25% reduction in OCT CRT from the baseline, with persistent or new IRF, SRF, or minimal or no change in visual acuity (less than five letters) after VEGF therapy [31,32].

### 2.2. PBMCs Isolation

Blood samples were drawn and PBMCs were isolated using a standard protocol for RNA extraction and purification. Briefly, blood was placed in BD Vacutainer CPT tubes containing Sodium Citrate and Ficoll™ Hypaque™ Solution (BD-Bioscience, Madrid, Spain). PBMCs were collected by centrifugation at 1800× *g* for 10 min at room temperature. Then, PBMCs were washed in a PBS -/- solution, centrifuged at 300× *g* for 10 min and quantified. Finally, PBMCs were stored at −80 °C after their resuspension in TRIzol reagent (Invitrogen, Madrid, Spain).

### 2.3. RNA Purification

Total RNA was extracted and purified according to published protocols [33]. Briefly, we isolated mRNA and miRNA using TRIzol (Invitrogen, Madrid, Spain) and the RNeasy mini-kit (Qiagen, Valencia, CA, USA). Samples were treated DNase I (Qiagen, Valencia, CA, USA) following the manufacturer’s instructions. Nucleic acid content was quantified with a Nanodrop spectrophotometer (Thermo Fisher Scientific, Waltham, MA, USA) and subjected to next-generation sequencing.

### 2.4. Next Generation Sequencing

Whole transcriptome sequencing was performed according to the manufacturer’s protocols and using their reagents (Illumina, San Diego, CA, USA) as described previously [33]. Briefly, an automated electrophoresis system (Experion; Bio-Rad, Hercules, CA, USA) was used to check the integrity and quality of the total. One μg mRNA was fragmented into approximately 200 base pair (bp) pieces by using divalent cations under elevated temperature. Cleaved RNA fragments were treated with reverse transcriptase and random primers to generate first strand cDNA. The second strand was obtained using DNA polymerase I and RNase H. cDNA fragments were end-repaired by Klenow DNA polymerase and T4 DNA polymerase. Then, cDNA was phosphorylated by T4 polynucleotide kinase and ligated indexing adapters (Illumina, Madrid, Spain). Adapter-tagged libraries were amplified by using PCR with DNA polymerase (Phusion; Finnzymes Reagents, Vantaa, Finland) and validated and quantified by electrophoresis and qPCR. Pools of 4–6 indexed libraries were mixed at equimolar ratios to yield a total oligonucleotide mixture concentration of 10 nM. Final libraries were sequenced in a HiSeq 1500 platform (Illumina, Madrid, Spain) to generate 2 × 125-bp paired-end reads.

### 2.5. Transcriptomic Analysis and Bioinformatics

For transcriptomic analyses, we used paired end raw reads data obtained from Illumina sequencing workflows. Firstly, data were cleaned of adapters and low-quality sequences based on reported quality scores. Mapping was performed using the Spliced Transcripts Alignment to a Reference (STAR) aligner (https://github.com/alexdobin/STAR accessed date 1 April 2021) [34]. Mapped reads were counted using FeatureCounts (http://subread.sourceforge.net/ accessed date 1 April 2021) over gene feature discarding multimapping reads [35]. The statistical analyses were performed on R using two different differential expression analysis packages: edgeR and DESeq2. Results were cross-checked between both methods to obtain a differential expressed gene set.

All sequenced samples were classified in the two defined patient groups (Good responders and Poor responders) to perform model contrasts. Gender classification was included in the statistical model as a confounding variable, not being used for contrast. RAW counts were normalized using the default methodology for each package, and zeros were excluded were less than 25% of samples had at least one count. Statistical results from model contrast were listed and filtered by *p*-value (<0.01) to study the most relevant alterations between groups. In addition, a wider filter was applied to analyze pathway enrichment by most altered genes (*p*-value < 0.05) using the Kyoto Encyclopedia of Genes and Genomes (KEGG) pathway database [36,37,38].

Micro RNA reads were obtained following Illumina sequencing protocols similarly to mRNA sequencing. Firstly, data were cleaned of adapters and low-quality sequences based on reported quality scores. After that, the miRDeep2 tool (https://github.com/rajewsky-lab/mirdeep2 accessed date 1 April 2021) was used for mapping, quantification and enrichment analysis over human described miRNAs in miRBase (https://www.mirbase.org accessed date 1 April 2021) and their precursors [39]. Differential miRNA expression analyses were performed over miRDeep2 normalized counts using the edgeR statistical package. The analysis setup was based on the same groups and criteria as mRNA differential expression analysis. Results were also filtered by p-value to identify the most relevant miRNAs altered between groups.

### 2.6. Data Mining and Feature Selection

All data mining and deep learning experiments were performed using open-source data mining tools (available from Weka Machine Learning 3, https://www.cs.waikato.ac.nz/ml/weka accessed date 1 April 2021). Firstly, an attribute evaluation algorithm was used to reduce gene sets used for classification modeling. That process was applied for both datasets (miRNA and mRNA) using two different approaches: model entropy minimization score for each attribute, and correlation-based feature selection algorithm. The resulting set reported by the correlation-based feature selection algorithm fulfills the condition of being the minimal attribute set without compromising model classification performance. For this study, we chose the area under the curve (AUC) of the receiver operating characteristic (ROC) to measure model classification performance. AUC was also used as a performance parameter in the data mining step. Signatures and their models were independently tested using 10-fold cross-validation techniques ensuring that no patient was present in both the training set and the test set.

## 3. Results

### 3.1. Cohort Description

Baseline characteristics of the AMD patients included in this study are presented in Table 1 and Supplementary Table S1. Fifty-nine patients were classified as “good responders”, or “poor responders” based on the morphological and functional criteria described in the “Methods” section. Prior to treatment, there were no differences between those groups in relation to age, sex, diabetes, hypertension, dyslipidemia, coronary disease, diet, tobacco or alcohol consumption, exposure to the sun, affected eye, intraocular pressure (IOP), central retinal thickness, macular cube volume, retinal pigment epithelial detachment, intra-retinal fluid, sub-retinal fluid, intra-retinal cysts, hemorrhage, exudation or fibrosis (Table 1). However, “poor responders” showed a higher proportion of RPE atrophy than “good responders” (*p* = 0.011) (Table 1), i.e., only 23% of the patients with RPE atrophy responded to treatment, while 64% of patients without atrophy showed improvements with ranibizumab.

### 3.2. Messenger RNA Expression Differences between Good Responders and Poor Responders

We performed RNA-Seq differential expression analysis on mRNA extracted from PBMCs of a patient with good or poor response to ranibizumab before treatment initiation.

The results of the comparison between those groups are shown in Table 2. Despite not reaching statistical significance when corrected for multiple testing, TOP25 genes were analyzed to construct a predictive classification model. Using a Random Forest classifier optimized by the meta classifier Random Committee the classifier shows a ROC curve with an AUC = 0.886. In addition, we performed pathway enrichment analysis using the KEGG database for exploratory purposes with sequences showing unadjusted *p* < 0.05. Interestingly, we found pathways related to inflammatory cytokines and NF-κB signaling (Supplementary Figure S1 and Supplementary Table S2), which supports the involvement of the immune system not only with the pathogenesis but also with the response to treatment.

### 3.3. Micro RNA Expression Differences between Good Responders and Poor Responders

We performed additional RNA-Seq differential expression analysis on micro RNA extracted from PBMCs of the same patients. The results of the comparison between good and poor responders are shown in Table 3.

Using mirPathv3.0-assisted data analysis [40] with the TOP25 miRNAs, we identified numerous pathways regulated by this set of miRNAs, including thyroid hormone signaling, TGF-β signaling, Hippo and adherent signaling, endocytosis and others. Noteworthy, this miRNA set also regulates important AMD-related genes such VEGF-A, PDGFA and PDGFRA involved in VEGF-signaling pathway, ECM-receptor interaction, cytokine-receptor interaction and cell cycle (Supplementary Figures S2–S6 and Supplementary Table S3). These results also support the involvement of relevant pathways, common to the immune system and the retina, with the response to treatment. Following the same strategy as with the mRNA data, we selected the TOP25 miRNAs to construct a predictive classification model based on Differential Expression (DE)-based classifiers. Using a Sequential Minimal Optimization (SMO)-based classifier we obtained a ROC curve with an AUC = 0.826.

### 3.4. Classification Model from mRNA Data

The whole transcriptome analysis (RNA-Seq) method allows measuring the expression levels of all the transcripts simultaneously. With that information, it is possible to develop different classification algorithms. Thus, there are several types of classifiers described in the literature. DE-based classifiers take into account the difference in the expression level of genes and they are often useful to discover disease biomarkers. For RNA-Seq data, this is typically done by fitting the distribution of the genes to negative binomial linear models and applying a likelihood ratio statistical test.

In general, the first step for a classification process is a feature selection step by pre-processing the data. This is done to reduce the computational complexity associated with the amount of data obtained by RNA-Seq. In addition, not all expressed genes may be relevant for a specific classification task. In fact, using uninformative features may even decrease the accuracy of the classifier by model overfitting.

However, since we did not observe any differentially expressed gene, between Good responders and Poor responders, after multiple testing corrections, we decided to investigate other classifiers based on entropy reduction.

**Figure 1:**
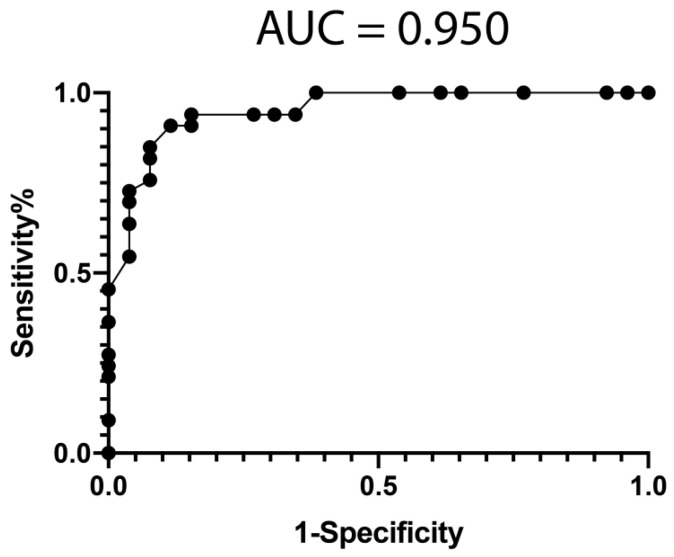
Classification model performance from informative mRNA data. A ROC was constructed with expression data from 10 mRNAs (ENSG00000249572, ENSG00000176531, ENSG00000240350, ENSG00000161298, ENSG00000049239, ENSG00000226479, ENSG00000198056, ENSG00000104450, ENSG00000156510, and ENSG00000158106) from the 59 patients. The classification model was built using a Random Forest classifier optimized by the meta classifier Random Committee, included in the WEKA suite. AUC = Area under the curve.

To reduce the number of features to use, we performed a feature selection procedure (see Methods), using the Weka suite [41], on mRNA sequencing data from PBMCs obtained from the studied cohort. This process resulted in a significant reduction of features (>1000-fold). We then selected the 10 most informative mRNAs (ENSG00000249572, ENSG00000176531, ENSG00000240350, ENSG00000161298, ENSG00000049239, ENSG00000226479, ENSG00000198056, ENSG00000104450, ENSG00000156510, and ENSG00000158106) to obtain a predictive classification model for treatment response, using a Random Forest classifier optimized by the meta classifier Random Committee. The performance of the classifier is represented with a ROC curve (AUC = 0.950) in Figure 1.

### 3.5. Classification Model from miRNA Data

**Figure 2:**
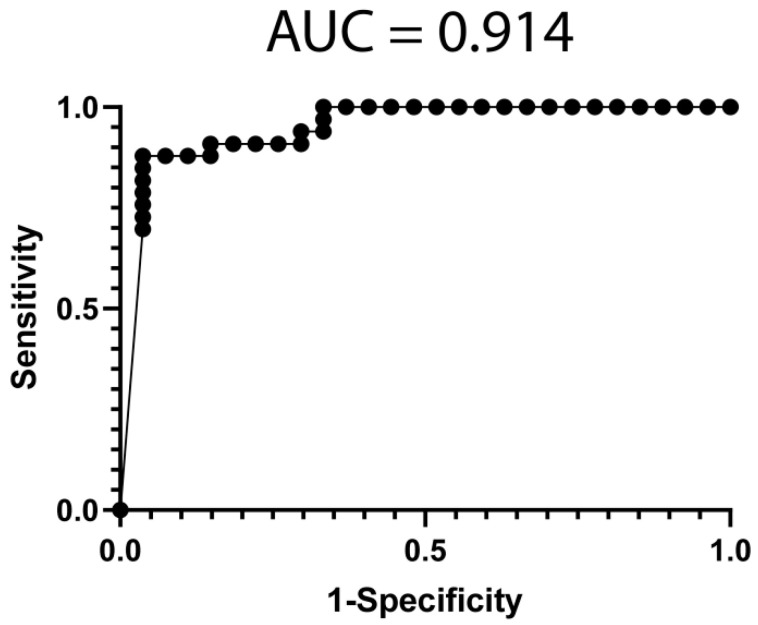
Classification model performance from informative miRNA data. A ROC was constructed with expression data from 18 miRNAs (hsa-miR-1284, hsa-miR-185-5p, hsa-miR-20a-5p, hsa-miR-210-5p, hsa-miR-3127-5p, hsa-miR-3149, hsa-miR-34c-5p, hsa-miR-511-5p, hsa-miR-548ah-3p, hsa-miR-551b-5p, hsa-miR-579-3p, hsa-miR-615-5p, hsa-miR-6786-3p, hsa-miR-6798-3p, hsa-miR-6813-5p, hsa-miR-6850-3p, hsa-miR-6875-5p and hsa-miR-6889-5p) from the 59 patients. The classification model was built using a SMO-based classifier, included in the WEKA suite. AUC = Area under the curve.

We performed feature selection (see Methods) on miRNA sequencing data from PBMCs obtained from the studied cohort. We selected the 18 most informative miRNAs (hsa-miR-1284, hsa-miR-185-5p, hsa-miR-20a-5p, hsa-miR-210-5p, hsa-miR-3127-5p, hsa-miR-3149, hsa-miR-34c-5p, hsa-miR-511-5p, hsa-miR-548ah-3p, hsa-miR-551b-5p, hsa-miR-579-3p, hsa-miR-615-5p, hsa-miR-6786-3p, hsa-miR-6798-3p, hsa-miR-6813-5p, hsa-miR-6850-3p, hsa-miR-6875-5p and hsa-miR-6889-5p) to obtain a predictive classification model for treatment response, using a Sequential Minimal Optimization (SMO)-based classifier. The performance of the classifier is represented with a ROC curve (AUC = 0.914) in Figure 2.

### 3.6. Classification Model from Combined mRNA and miRNA Data

**Figure 3:**
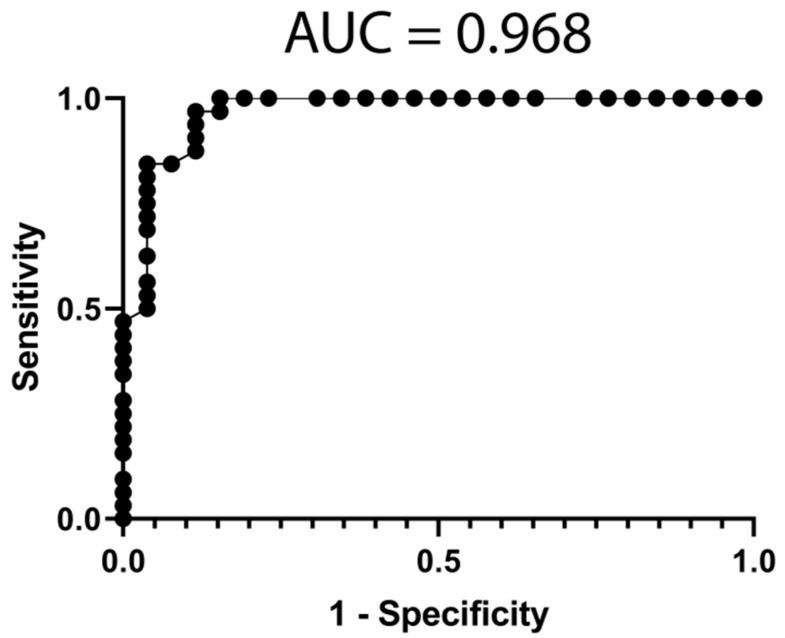
Classification model performance from informative mRNA and miRNA data. A ROC was constructed with expression data from mRNAs (ENSG00000249572, ENSG00000161298, ENSG00000226479 and ENSG00000198056) and 1 miRNAs (hsa-miR-20a-5p) from the 59 patients. The classification model was built using a Naïve Bayes classifier, included in the WEKA suite. AUC = Area under the curve.

In order to obtain an optimal classification model, we combined the most discriminative mRNAs (ENSG00000249572, ENSG00000161298, ENSG00000226479 and ENSG00000198056) with the most discriminative miRNA (hsa-miR-20a-5p) using an adjusted naïve Bayes classifier. The performance of the classifier for treatment response is represented with a ROC curve (AUC = 0.968) in Figure 3, which improved the mRNA model (AUC = 0.950, Figure 1) and the miRNA model (AUC = 0.914, Figure 2).

## 4. Discussion

Age-related macular degeneration (AMD) is a multifactorial disease comprising many risk factors. The main risk factors commonly associated with AMD are age, family history and cigarette smoke. Other risk factors include body mass index, ethnicity, gender, cardiovascular-related diseases, or dietary habits [42].

Anti-VEGF drugs are effective inhibitors of laser-induced CNV in animal models and they are the first line of therapy for wet AMD in humans [2,43]. There is no universal definition of treatment failure. Some professionals consider that the treatment has failed when fluid persists in the retina while others focus more on vision loss. In addition, treatment protocol, which can be altered by physicians for a variety of reasons, may also affect response. Furthermore, the “treat-and-observe” approach, often applied, may be related to some treatment failures.

In our study, 44% of the patients did not show satisfactory response to ranibizumab treatment after three months, based on our established criteria. This result, although elevated, is comparable with other real-world studies data [26,32,44,45]. When we compared the baseline characteristics of our cohort, based on the response to ranibizumab, we did not find any difference in systemic factors such age, sex, tobacco consumption, hypertension, etc., or baseline ocular measurements, such as intraocular pressure (IOP), central retinal thickness, macular cube volume, retinal pigment epithelial detachment, presence of fluids or fibrosis (Table 1). This is in agreement with previous studies where they did not find an association of these parameters with response to ranibizumab treatment [46,47], although some reports found an association of smoking history and hypertension with response to ranibizumab in exudative AMD [48].

Interestingly, although RPE atrophy was higher in the group with a poorer response, as it is seen regularly in the clinic [49,50,51], it did not show discriminatory power between groups (AUC = 0.64, *p* = 0.0544) in the present study.

In the past, we have used RNA-Seq technology successfully to elucidate the involvement of relevant pathways to the response to pharmacological treatment, in a complex ocular disease, through integrative pathway enrichment [33,52]. Other groups have analyzed monocytes using microarray-based transcriptomics, to reveal altered immune-related genes differences between AMD patients and age-matched controls [53].

In our cohort, due to the number of samples included, as well as the intrinsic variability associated with human samples, multiple comparisons correction failed to identify any significant differences in expression of mRNA or miRNA between good or poor responders. However, the exploratory analysis identified relevant genes/pathways that may be associated with the response to ranibizumab and warrant further research. Of note, poor responders showed an increase in angiogenic and pro-inflammatory cytokines mediated by nuclear factor kappa-B (NF-kB) signaling activation, which may be of relevance because systemic inflammatory factors have been associated with AMD pathogenesis [54,55]. However, the role in AMD onset, or ranibizumab response, of the four mRNAs and one miRNA comprising the best classification model remains unclear because the information in the literature is scarce. ENSG00000249572 (lnc-ADAMTS12-6) is a novel transcript affiliated with the long noncoding RNA (lncRNA) class, located in chromosome 5 of the human genome. Although functional characterization is still lacking for most lncRNAs, they have been implicated in several human diseases through diverse mechanisms. Thus, lncRNAs can bind to genomic DNA, regulating local chromatin structure in the nucleus. They can also regulate gene expression post-transcriptionally by interacting with mRNA translation proteins and miRNAs [56]. This transcript has been associated with lncRNA deregulation occurring in PBMCs from Juvenile myelomonocytic leukemia patients carrying a mutation in the NF1 gene [57] suggesting a role in immune regulation. ENSG00000161298 (ZNF382) belongs to the KRAB domain zinc finger transcription factor (KZNF) family which has been shown to regulate differentiation, proliferation and apoptosis processes by interacting with activating protein 1 (AP-1), Fos proto-oncogene (FOS), Jun proto-oncogen (JUN) and NF-kB signaling [58]. These interactions result in inhibition of these inflammatory pathways which is in agreement with the notion that poor responders, showing reduced levels of this anti-inflammatory transcription factor, may be displaying a pro-inflammatory profile in PBMCs gene expression. In addition, ZNF382 upregulation reduces the expression of metalloproteinases, such as MMP1 [58], that are dysregulated in AMD [59]. ENSG00000226479 (TMEM185B) is a widely expressed protein coding gene belonging to the TMEM family which gathers proteins of mostly unknown functions. They usually have roles related to proliferation and some of them have been defined as potential prognostic biomarkers for lung cancer [60]. Interestingly, this member has been associated with Bare Lymphocyte Syndrome, type II, an immune system disease categorized as a form of combined immunodeficiency. Lastly, ENSG00000198056 encodes a small subunit of the Primase protein (PRIM1) involved in the replication of the DNA and cellular proliferation. This primase regulates critical processes in the retina since mutations on this primase promote apoptosis of retinal neurons through the activation of p53 and DNA damage checkpoints [61] and its function has been associated with the development of inherited retinal degenerations, such as recessive Retinitis Pigmentosa [62]. The fifth member of the best classification model is a miRNA (hsa-miR-20a-5p) expressed in the retina [63], able to regulate a high number of genes (Supplementary Table S4), including genes related to the regulation of cell proliferation [64], immune system [65] and apoptosis [66,67]. Interestingly, hsa-miR-20a-5p represses endothelial cell migration, in response to VEGF, regulating MKK3 and p38 MAP kinase activation [68,69].

In the present year, a machine-learning method for the classification of patients, at the first visit, requiring high or low treatment demand was presented showing promising results (AUCs ranging 0.76–0.79, 10-fold cross-validated) using morphological features from OCT [70]. Recently, a method to predict, retrospectively, the response to 2-year treatment of ranibizumab in AMD patients was reported [71]. This method relayed in a model-based meta-marker defined by a composite score of 7 baseline characteristics to categorize the response to ranibizumab. These seven characteristics were comprised of: age, central retinal lesion thickness, visual acuity, presence of cysts, type of CNV and PED and leakage sizes. In our cohort, we measured the response after 3 monthly injections, and we were not capable of predicting that response accurately based on any of the seven characteristics reported. The only statistically significant characteristic between Good responders and Poor responders in our cohort was the presence of RPE atrophy, which did not result as discriminatory among groups.

The multifactorial nature of the disease, the recent advances in molecular measurements (RNA-Seq) and computational techniques (machine learning-based) lead us to hypothesize that PBMC global transcriptomic analysis may provide further insights into the response to ranibizumab in AMD patients. In recent years, transcriptomics of peripheral blood mononuclear cells has been also used to capture the variability of the immune system in autoimmune or liver diseases, among others [72,73,74]. In our study, we obtained moderate results using differential expression-based methods (AUC = 0.886 with a 25-member signature for mRNA data and AUC = 0.826 with a 25-member signature for miRNA data). These results were somehow expected due to the high variability usually obtained when working with human samples [75]. For that reason, we decided to perform feature selection with entropy reduction-based which resulted in better models (AUC > 0.9 for mRNA and miRNA data) using fewer features, especially when we combined mRNA and miRNA data.

We acknowledge, however, that our study has some limitations. First, we analyzed the response of the patients after three months of treatment. Our reasoning was that, although ranibizumab may be effective after longer periods, it is often after these three months that the physicians decide whether to continue or modify the treatment or the regimen. Second, a similar study with a larger cohort in terms of the number of patients would be beneficial to validate the results. In order to reduce the possibility of overfitting, we performed 10-fold cross-validation to our feature selection and model construction. In addition, even though the cohort size is large enough to reach significant conclusions with the machine learning techniques we used, we believe that these results would need to be replicated in other cohorts in the future. Given the disparity of regimens found in the literature [76,77], it is difficult to find comparable studies with available sequencing data. For these reasons, we plan to replicate a similar study in the future to include data, not only from mRNA and miRNA but also from DNA methylome, from a new study we are currently conducting.

In summary, here we describe a signature comprised of four mRNAs and one miRNA in PBMCs from AMD patients that allowed us to predict, retrospectively, a successful response to ranibizumab before the start of the treatment, with good accuracy. Furthermore, we consider that machine learning classifiers based on mRNA and miRNA from PBMCs, especially in combination with other methods, such as specific baseline characteristics, may improve the prediction of patients with insufficient response to ranibizumab and help establish patient-specific treatment plans at the first visit.

## Figures and Tables

**Table 1:** Demographics and baseline characteristics.

		All	Poor Responders	Good Responders	*p*-Value
	Age (years, mean ± SD)	78.03 ± 1.055	80.04 ± 1.379	76.45 ± 1.504	0.092
	Sex (Male/Female)	24/35	8/18	16/17	0.193
	Tobacco (yes/no)	23/36	8/18	15/18	0.292
	ETDRS (letters)	63.61 ± 1.131	62.73 ± 1.791	64.30 ± 1.464	0.495
OCT	Central Retinal Thickness, mean±SD (µm)	329.18 ± 15.495	325.2 ± 28.09	332.4 ± 17.7	0.820
	Macular cube volume (µm^3^)	10.333 ± 0.176	10.37 ± 0.3137	10.30 ± 0.2004	0.839
	Retinal pigment epithelial detachment (yes/no)	43/16	20/6	23/10	0.571
	Intra-retinal fluid (yes/no)	48/11	22/4	26/7	0.740
	Sub-retinal fluid (yes/no)	43/16	17/9	26/7	0.377
	Intra-retinal cysts (yes/no)	35/24	16/10	19/14	0.795
Fundoscopy	Hemorrhage (yes/no)	28/31	15/11	13/20	0.196
	Exudation (yes/no)	8/51	2/24	6/27	0.446
	Atrophy (yes/no)	13/46	10/16	3/30	0.011
	Fibrosis (yes/no)	4/55	2/24	2/31	1.000
AGF	Location (Sub-/Yuxta-/Extra-Foveal)	16/35/5	9/16/0	7/19/5	0.085
	Size (mm)	1.03 ± 0.130	1.193 ± 0.2181	0.9150 ± 0.1585	0.297
	Pattern (O/PC/MC)	26/22/8	14/9/3	12/13/5	0.577

*p* values were calculated using Fisher’s exact test. AGF pattern: O = Occult; PC = predominantly classic; MC: Minimally classic.

**Table 2:** Differentially expressed mRNAs in PBMCs from Good responders and Poor responders.

Ensembl_ID	BaseMean	Log2FC	*p* Value	Symbol	Name
ENSG00000273727	3.8	−1.65	4.10 × 10^−5^	U1	U1 spliceosomal RNA
ENSG00000235621	9.8	0.82	1.16 × 10^−4^	LINC00494	long intergenic non-protein coding RNA 494
ENSG00000158106	25.0	0.54	1.67 × 10^−4^	RHPN1	rhophilin Rho GTPase binding protein 1
ENSG00000215012	75.3	0.25	2.13 × 10^−4^	RTL10	retrotransposon Gag like 10
ENSG00000249572	1.8	−1.31	2.41 × 10^−4^	N/A	novel transcript
ENSG00000233913	23.6	−1.95	3.18 × 10^−4^	RPL10P9	ribosomal protein L10 pseudogene 9
ENSG00000260766	12.5	0.49	3.98 × 10^−4^	N/A	N/A
ENSG00000160307	18.5	−1.57	4.40 × 10^−4^	S100B	S100 calcium binding protein B
ENSG00000226581	1.1	1.62	5.17 × 10^−4^	LINC02848	long intergenic non-protein coding RNA 2848
ENSG00000204345	1.3	−1.40	5.83 × 10^−4^	CD300LD	CD300 molecule like family member d
ENSG00000174473	1.7	1.56	7.12 × 10^−4^	GALNTL6	polypeptide N-acetylgalactosaminyltransferase like 6
ENSG00000211789	14.2	0.77	7.23 × 10^−4^	TRAV12-2	T cell receptor alpha variable 12-2
ENSG00000205710	11.6	−0.80	7.28 × 10^−4^	C17orf107	chromosome 17 open reading frame 107
ENSG00000227309	3.3	−0.95	7.79 × 10^−4^	RPL31P19	ribosomal protein L31 (RPL31) pseudogene
ENSG00000154723	43.8	−0.26	7.79 × 10^−4^	ATP5PF	ATP synthase peripheral stalk subunit F6
ENSG00000107968	221.6	−0.29	7.81 × 10^−4^	MAP3K8	mitogen-activated protein kinase kinase kinase 8
ENSG00000211880	6.4	0.70	7.90 × 10^−4^	TRAJ9	T cell receptor alpha joining 9
ENSG00000258511	2.1	1.13	7.95 × 10^−4^	LINC02295	long intergenic non-protein coding RNA 2295
ENSG00000235361	0.8	−1.67	9.34 × 10^−4^	N/A	novel transcript, antisense to ABR
ENSG00000226430	1.7	1.29	1.03 × 10^−3^	USP17L7	ubiquitin specific peptidase 17 like family member 7
ENSG00000197353	6.9	−0.94	1.04 × 10^−3^	LYPD2	LY6/PLAUR domain containing 2
ENSG00000180758	18.7	0.39	1.06 × 10^−3^	GPR157	G protein-coupled receptor 157
ENSG00000211575	0.9	−1.60	1.19 × 10^−3^	MIR760	microRNA 760
ENSG00000272666	12.3	−0.75	1.26 × 10^−3^	KLHDC7B-DT	KLHDC7B divergent transcript
ENSG00000156510	15.3	0.55	1.29 × 10^−3^	HKDC1	hexokinase domain containing 1
ENSG00000007038	5.5	0.88	1.32 × 10^−3^	PRSS21	serine protease 21
ENSG00000254088	5.4	0.61	1.33 × 10^−3^	SLC2A3P4	solute carrier family 2 member 3 pseudogene 4
ENSG00000281106	23.3	0.71	1.58 × 10^−3^	TMEM272	transmembrane protein 272
ENSG00000180822	67.2	0.28	1.58 × 10^−3^	PSMG4	proteasome assembly chaperone 4
ENSG00000236709	7.6	−1.10	1.61 × 10^−3^	DAPK1-IT1	DAPK1 intronic transcript 1

**Table 3:** Differentially expressed miRNAs in PBMCs from Good responders and Poor responders.

miRNA	BaseMean	Log2FC	*p* Value
hsa-miR-3614-5p	11.3	3.49	1.42 × 10^−3^
hsa-miR-423-5p	1779.1	10.80	2.37 × 10^−3^
hsa-miR-20a-5p	865.9	9.76	3.16 × 10^−3^
hsa-miR-30c-1-3p	24.6	4.62	5.19 × 10^−3^
hsa-miR-1249-5p	3.0	1.58	5.32 × 10^−3^
hsa-miR-3605-3p	7.5	2.91	6.96 × 10^−3^
hsa-miR-320b	22.2	4.48	7.55 × 10^−3^
hsa-miR-320b-2	22.7	4.51	8.09 × 10^−3^
hsa-miR-25-5p	11.8	3.56	9.93 × 10^−3^
hsa-miR-34c-5p	4.7	2.22	1.36 × 10^−2^
hsa-miR-6813-5p	2.8	1.48	1.67 × 10^−2^
hsa-miR-30a-5p	225.9	7.82	1.73 × 10^−2^
hsa-miR-132-3p	25.0	4.64	1.76 × 10^−2^
hsa-miR-642a-5p	4.3	2.10	1.77 × 10^−2^
hsa-miR-212-3p	6.9	2.78	1.97 × 10^−2^
hsa-miR-181b-5p	1721.2	10.75	1.98 × 10^−2^
hsa-miR-3127-5p	2.8	1.47	2.00 × 10^−2^
hsa-miR-1273h-5p	6.4	2.68	2.11 × 10^−2^
hsa-miR-181b-5p-2	1795.9	10.81	2.19 × 10^−2^
hsa-miR-652-5p	24.7	4.63	2.51 × 10^−2^
hsa-let-7i-5p	5009.0	12.29	2.51 × 10^−2^
hsa-miR-369-3p	20.0	4.32	2.59 × 10^−2^
hsa-miR-6511a-3p	4.7	2.24	2.62 × 10^−2^
hsa-miR-6511a-3p-2	4.7	2.24	2.62 × 10^−2^
hsa-miR-6511a-3p-3	4.7	2.24	2.62 × 10^−2^
hsa-miR-6511a-3p-4	4.7	2.24	2.62 × 10^−2^
hsa-miR-223-5p	93.8	6.55	2.69 × 10^−2^
hsa-miR-2110	14.5	3.85	2.84 × 10^−2^
hsa-miR-190a-5p	32.2	5.01	2.91 × 10^−2^
hsa-miR-487a-3p	9.7	3.28	3.27 × 10^−2^

## Supplementary Materials

The following are available online at https://www.mdpi.com/article/10.3390/jpm11121329/s1, Figure S1: NF-κB/cytokine signaling pathway members regulated by TOP25 differentially expressed mRNAs: TOP25 miRNAs (from Table 2) were analyzed for exploratory KEGG pathway enrichment. Green: Genes downregulated in good responders vs poor responders. Red: Genes upregulated in good responders vs poor responders. Figure S2: Endocytosis regulation by TOP25 differentially expressed miRNAs: TOP25 miRNAs (from Table 3) were analyzed with DIANA-miRPath v3.0 for exploratory KEGG pathway enrichment. Yellow: Genes regulated by 1 miRNA. Orange: Genes regulated by 2 or more miRNAs. Figure S3: Gap junction members regulated by TOP25 differentially expressed miRNAs: TOP25 miRNAs (from Table 3) were analyzed with DIANA-miRPath v3.0 for exploratory KEGG pathway enrichment. Yellow: Genes regulated by 1 miRNA. Orange: Genes regulated by 2 or more miRNAs. Figure S4:Hippo signaling pathway members regulated by TOP25 differentially expressed miRNAs: TOP25 miRNAs (from Table 3) were analyzed with DIANA-miRPath v3.0 for exploratory KEGG pathway enrichment. Yellow: Genes regulated by 1 miRNA. Orange: Genes regulated by 2 or more miRNAs. Figure S5: Thyroid hormone signaling pathway members regulated by TOP25 differentially expressed miRNAs: TOP25 miRNAs (from Table 3) were analyzed with DIANA-miRPath v3.0 for exploratory KEGG pathway enrichment. Yellow: Genes regulated by 1 miRNA. Orange: Genes regulated by 2 or more miRNAs. Figure S6:Pathways-in-Cancer KEGG members regulated by TOP25 differentially expressed miRNAs: TOP25 miRNAs (from Table 3) were analyzed with DIANA-miRPath v3.0 for exploratory KEGG pathway enrichment. Yellow: Genes regulated by 1 miRNA. Orange: Genes regulated by 2 or more miRNAs., Table S1: Altered KEGG pathways by TOP25 mRNAs. TOP25 mRNAs differentially expressed were subjected to exploratory analysis. Table S2: Altered KEGG pathways by TOP25 miRNAs. TOP25 miRNAs differentially expressed were subjected to exploratory analysis. Table S3: List of putative genes regulated by hsa-miR-20a-5p according to TargetScan v7.2 database.
