# Supplementary material for: Predictive Biomarkers of Age-Related Macular Degeneration Response to Anti-VEGF Treatment
Source: J Pers Med. 2021 Dec 8;11(12):1329. doi: 10.3390/jpm11121329 (PMC8706948; doi:10.3390/jpm11121329)
Supplement: Supplementary file 1 [file jpm-11-01329-s001.zip › jpm-1452968-supplementary/Figure S1.pptx]

## Slide 1
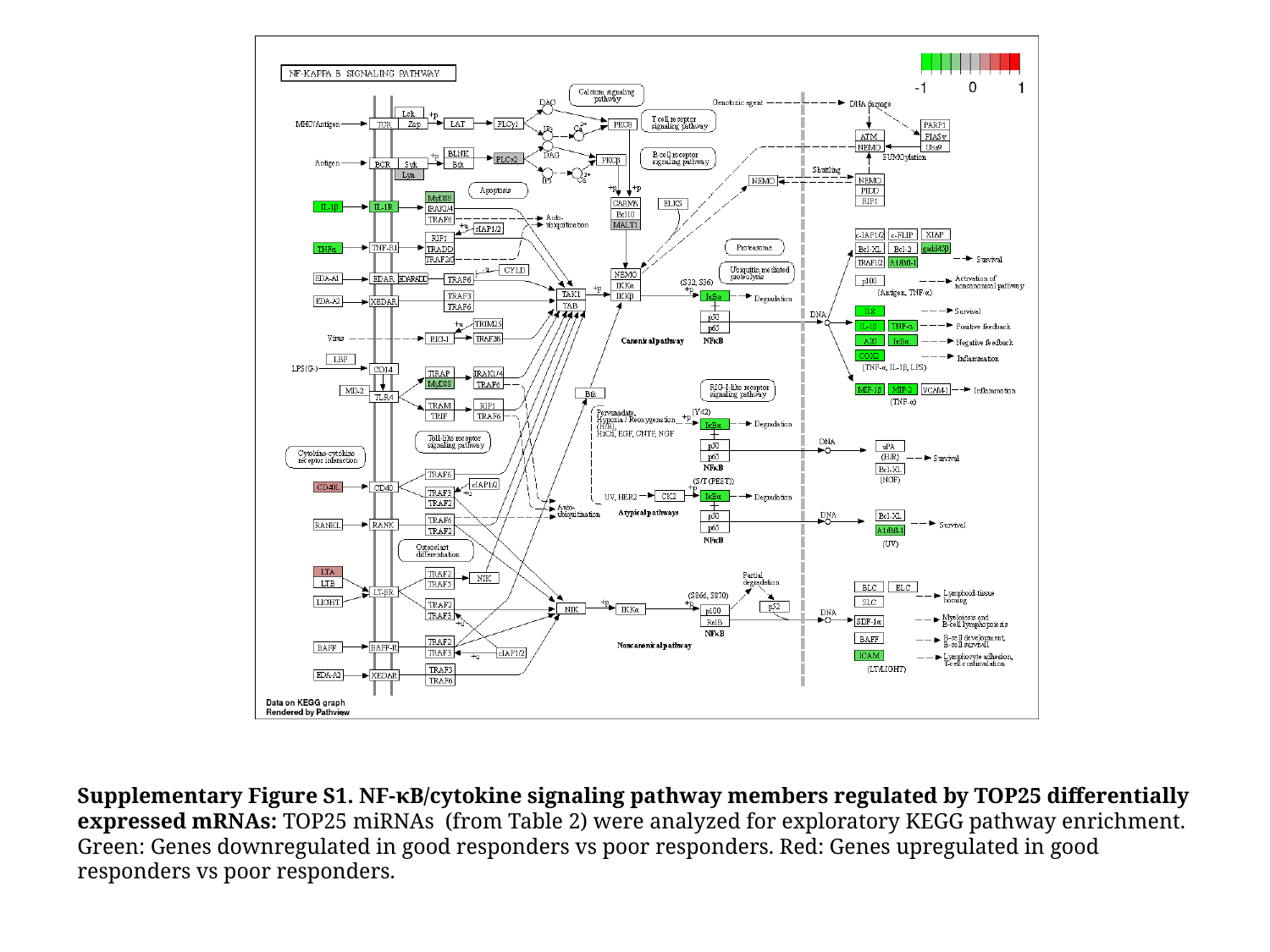

Supplementary Figure S1. NF-κB/cytokine signaling pathway members regulated by TOP25 differentially expressed mRNAs: TOP25 miRNAs (from Table 2) were analyzed for exploratory KEGG pathway enrichment. Green: Genes downregulated in good responders vs poor responders. Red: Genes upregulated in good responders vs poor responders.
