# Supplementary material for: Predictive Biomarkers of Age-Related Macular Degeneration Response to Anti-VEGF Treatment
Source: J Pers Med. 2021 Dec 8;11(12):1329. doi: 10.3390/jpm11121329 (PMC8706948; doi:10.3390/jpm11121329)
Supplement: Supplementary file 1 [file jpm-11-01329-s001.zip › jpm-1452968-supplementary/Figures S2-S6.pptx]

## Slide 1
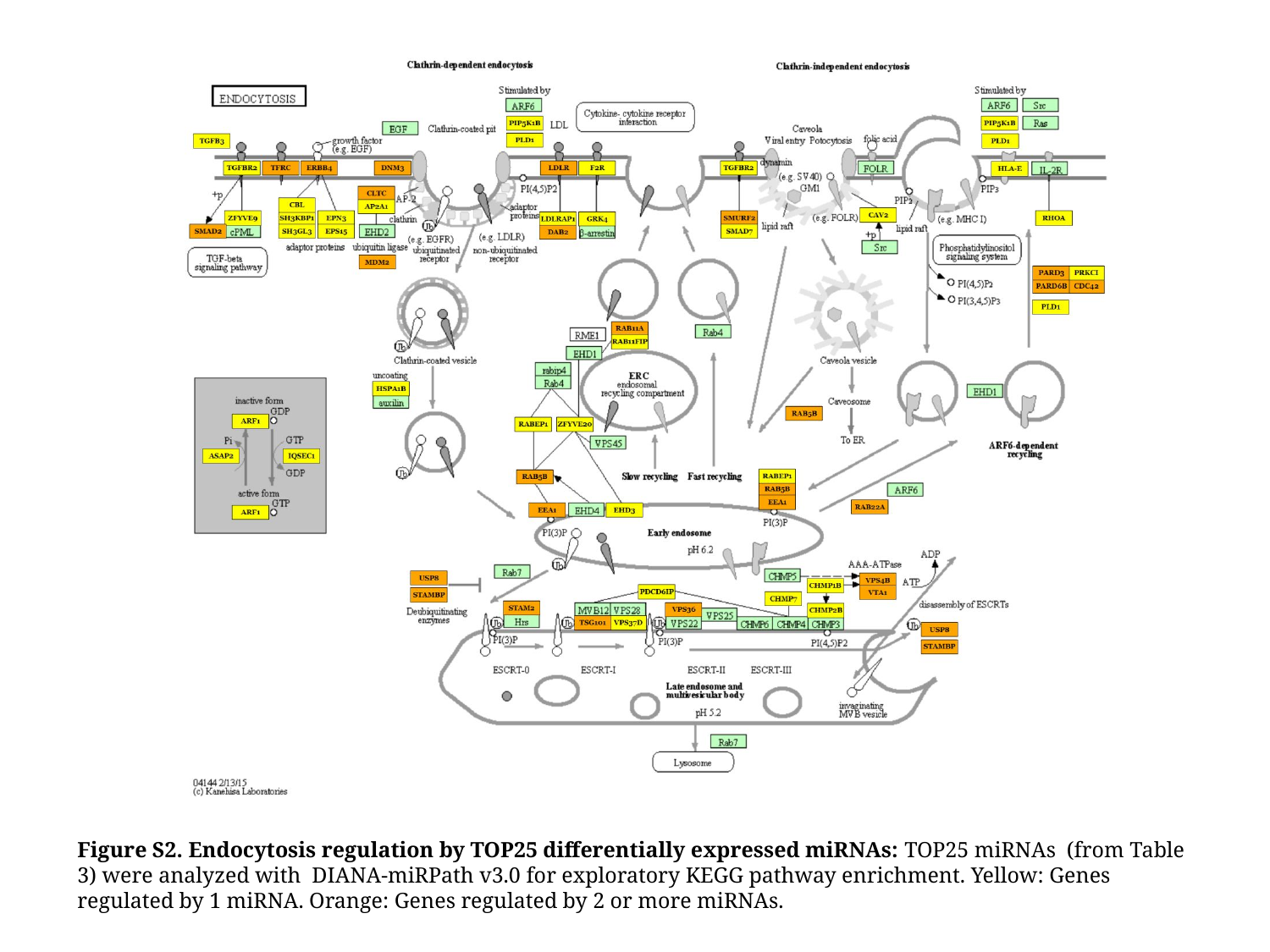

Figure S2. Endocytosis regulation by TOP25 differentially expressed miRNAs: TOP25 miRNAs (from Table 3) were analyzed with DIANA-miRPath v3.0 for exploratory KEGG pathway enrichment. Yellow: Genes regulated by 1 miRNA. Orange: Genes regulated by 2 or more miRNAs.

## Slide 2
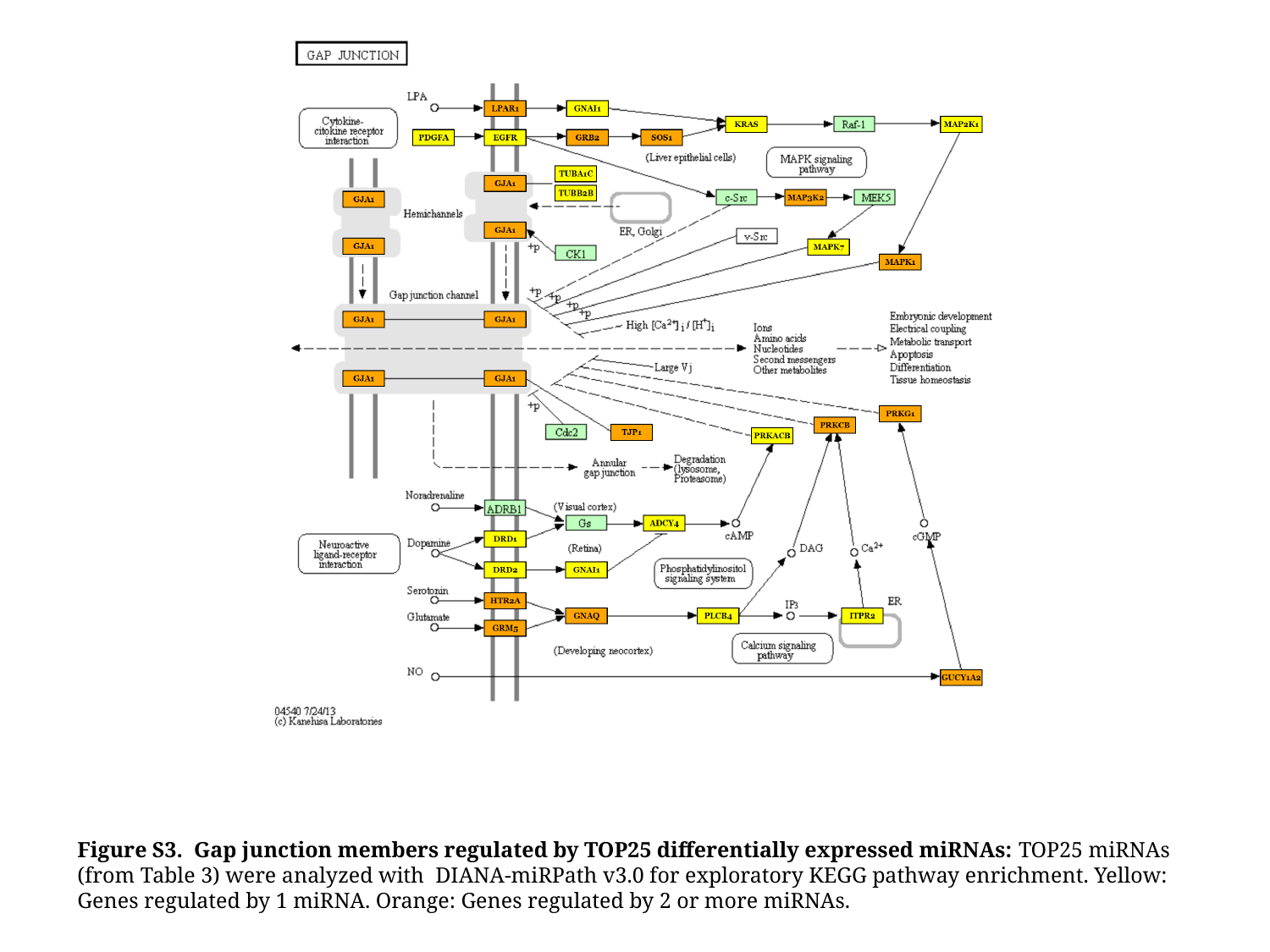

Figure S3. Gap junction members regulated by TOP25 differentially expressed miRNAs: TOP25 miRNAs (from Table 3) were analyzed with DIANA-miRPath v3.0 for exploratory KEGG pathway enrichment. Yellow: Genes regulated by 1 miRNA. Orange: Genes regulated by 2 or more miRNAs.

## Slide 3
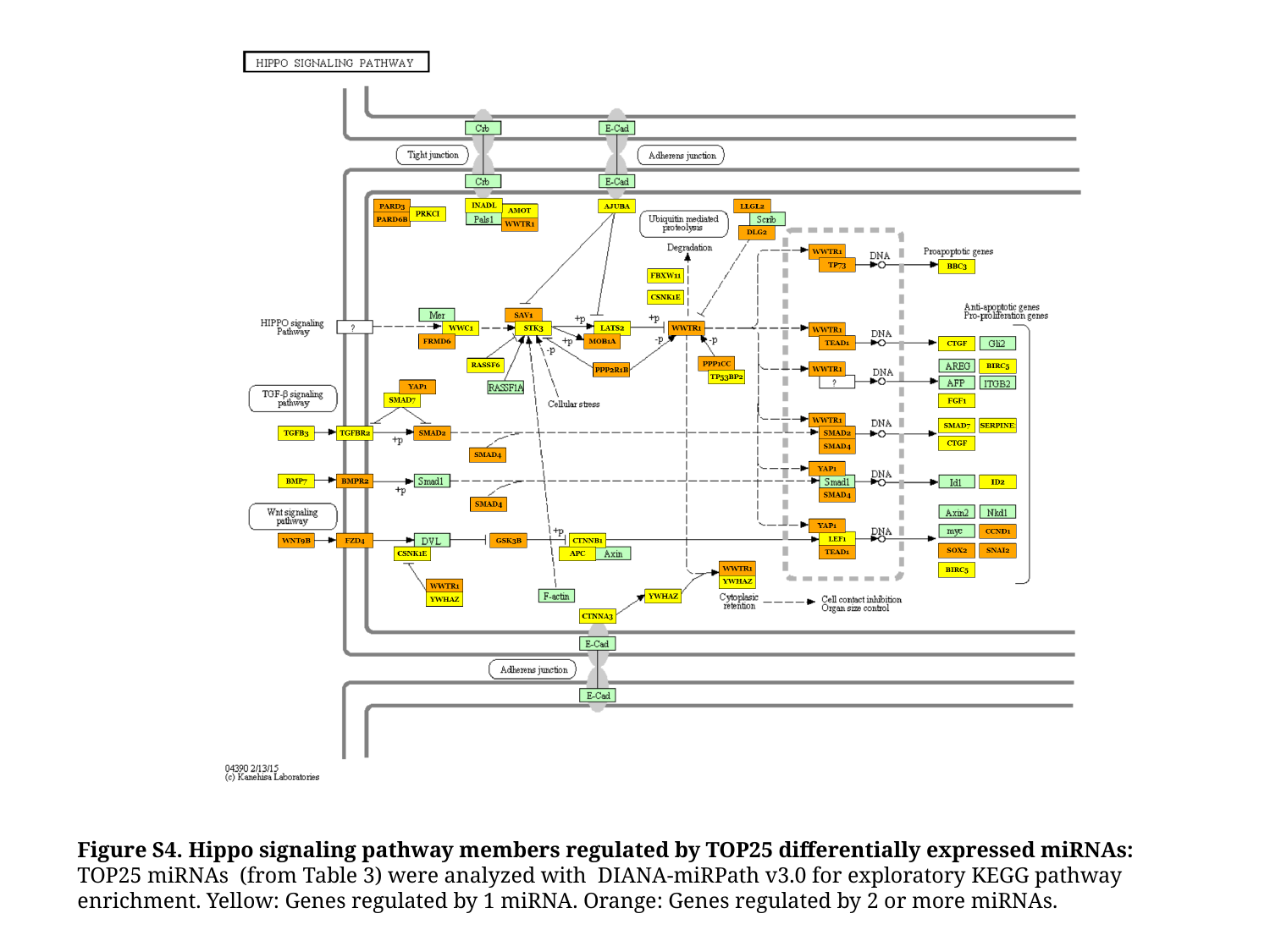

Figure S4. Hippo signaling pathway members regulated by TOP25 differentially expressed miRNAs: TOP25 miRNAs (from Table 3) were analyzed with DIANA-miRPath v3.0 for exploratory KEGG pathway enrichment. Yellow: Genes regulated by 1 miRNA. Orange: Genes regulated by 2 or more miRNAs.

## Slide 4
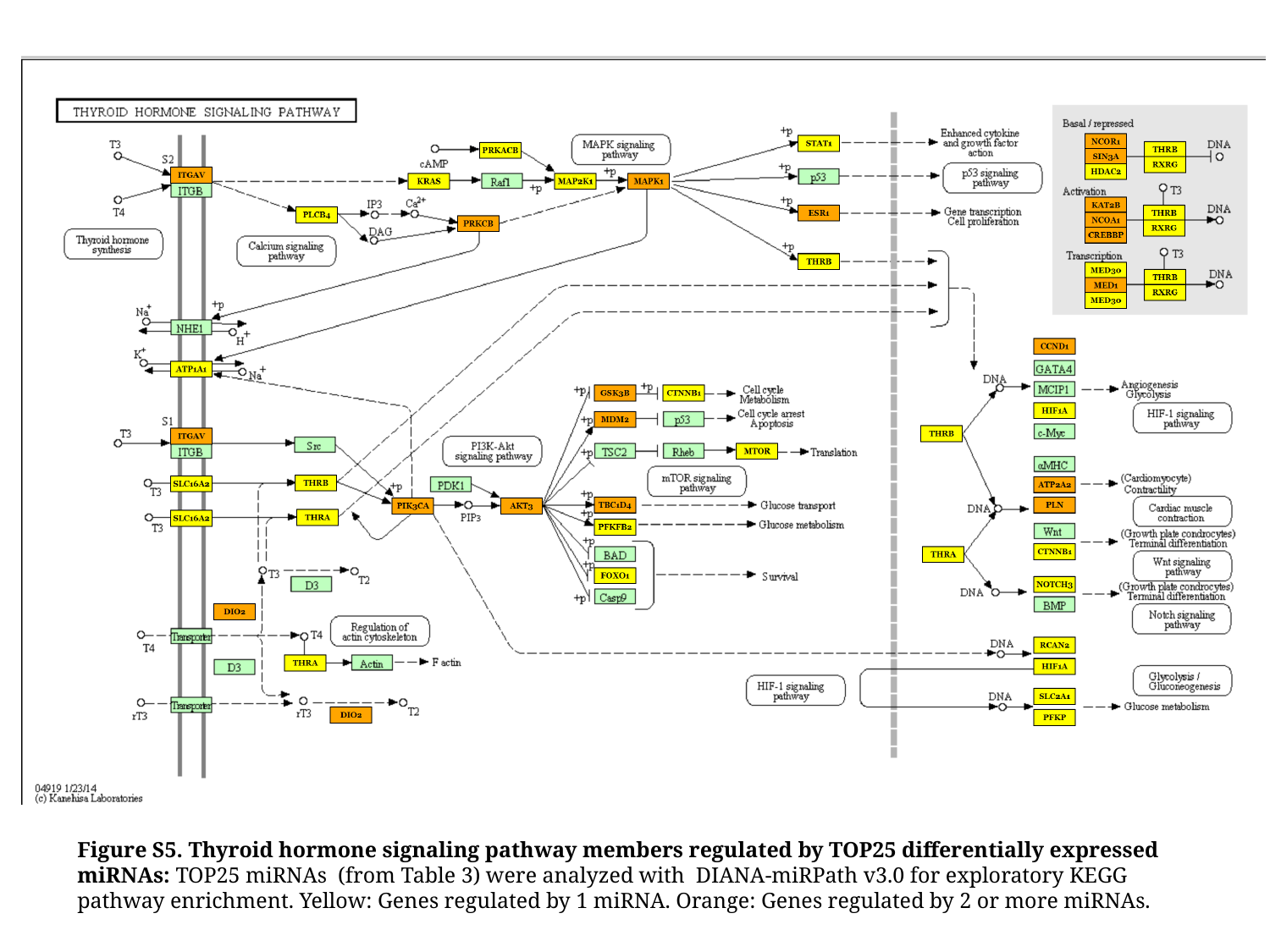

Figure S5. Thyroid hormone signaling pathway members regulated by TOP25 differentially expressed miRNAs: TOP25 miRNAs (from Table 3) were analyzed with DIANA-miRPath v3.0 for exploratory KEGG pathway enrichment. Yellow: Genes regulated by 1 miRNA. Orange: Genes regulated by 2 or more miRNAs.

## Slide 5
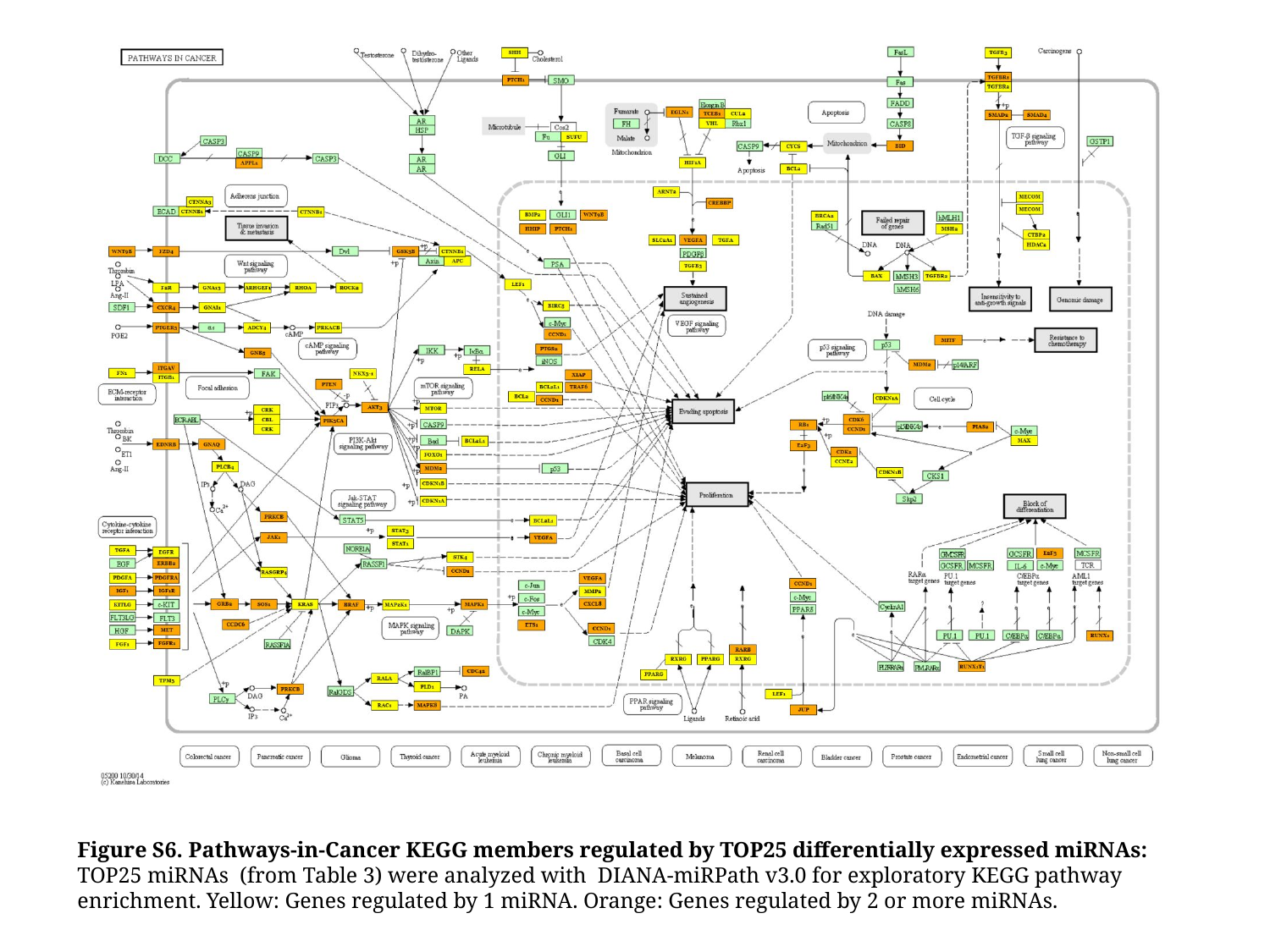

Figure S6. Pathways-in-Cancer KEGG members regulated by TOP25 differentially expressed miRNAs: TOP25 miRNAs (from Table 3) were analyzed with DIANA-miRPath v3.0 for exploratory KEGG pathway enrichment. Yellow: Genes regulated by 1 miRNA. Orange: Genes regulated by 2 or more miRNAs.
